# Food addiction and impulsivity in adolescents: A cross-sectional survey of 360 cases

**DOI:** 10.1192/j.eurpsy.2023.1536

**Published:** 2023-07-19

**Authors:** M. Chaabane, D. Ben Touhemi, K. Chiha, W. Kammoun, J. Boudabous, I. Hajkacem, H. Ayadi, K. Khemakhem, Y. Moalla

**Affiliations:** Department of Child Psychiatry, Hedi Chaker Hospital, Sfax, Tunisia

## Abstract

**Introduction:**

Impulsive personality Traits have been highly associated with both alcohol abuse and drug addiction, but have been accorded little attention in the context of food addiction.

**Objectives:**

To study the relationship between impulsivity and food addiction in school-aged adolescents.

**Methods:**

It is a cross-sectional, descriptive and analytical survey, conducted in a sample of secondary school students, randomly collected in 6 schools in the region of Sfax during February 2022. A pre established form of 33 questions, including socio- demographic and family information was used. Impulsivity was evaluated by the Barratt Impulsivity Scale (BIS-11; Patton et al., 1995). The BIS is a 30-item questionnaire that measures impulsivity along the following dimensions: cognitive, motor, and non-planning. The validated arabic version was used. *

The 25-item Yale Food Addiction Scale (YFAS), validated in Arabic, was used to assess food addiction in adolescents.

**Results:**

Our sample consisted of 360 adolescents, with an average age of 16.62 years, being male in 52.2% and with a low to medium socio-economic level in 72.7% of them.

A total of 20% of the adolescents showed a tendency to impulsivity, 23.6% had impulse control disorder.

The food addiction score of our sample ranged from 0 to 56 with an average of 16.37 ± 12.380.

The average food addiction score for adolescents with impulse control disorder was 20.21 ±14.819 while the average food addiction score for adolescents without impulse control disorder was 15.18 ± 11.291.

Food addiction was strongly associated with impulsivity (p < 0.001).In particularly, Non-planning impulsivity was most strongly correlated with food addiction (p < 0.001, r ꞊ 0.252)

**Conclusions:**

Impulsivity, commonly related to addictive substance use behaviors, may be a significant risk factor for food addiction. Early monitoring of impulse control disorder may help to reduce addictive food consumption.

**Disclosure of Interest:**

None Declared

